# Effects of phytase and protease supplementation, with and without considering their nutritional matrix values, on the productive performance of laying hens during the late‑lay period

**DOI:** 10.1016/j.vas.2026.100864

**Published:** 2026-09-11

**Authors:** Elahe Maghami, Sina Payvastegan, Mohsen Daneshyar, Touba Nadri

**Affiliations:** Department of Animal Science, Faculty of Agricultural Sciences, Urmia University, Urmia, Iran

**Keywords:** Laying hens, Nutritional matrix, Phytase, Productive performance, Protease

## Abstract

This study investigated the effects of phytase and protease supplementation, with or without considering their nutritional matrix values, on 384 Hy-Line W-80 laying hens at 63 weeks of age. The experiment was conducted as a 2×2×2 factorial arrangement in a completely randomized design with eight treatments and six replicates of eight birds each. The trial lasted 10 weeks, during which performance parameters, internal egg quality, eggshell traits, selected blood metabolites and ovary status were evaluated. Results showed that protease supplementation in diets without phytase increased (*P*<0.05) egg production, and egg production was also higher (*P*<0.01) with on-top enzyme supplementation. Egg weight, egg mass, and feed conversion ratio were not affected (*P*>0.05) by any main or interaction effects. Protease supplementation increased (*P*<0.05) the Haugh unit and yolk color score. The three-way interaction of phytase, protease, and consideration of their nutritional matrix significantly affected eggshell thickness and calcium content. When phytase was used alone, considering its nutritional value increased eggshell thickness (*P*<0.01) and calcium content (*P*<0.05) compared with on-top supplementation. In addition, matrix-based phytase supplementation improved (*P*<0.05) shell strength. Eggshell phosphorus concentration was also higher (*P*<0.05) in diets supplemented with protease in the absence of phytase. Total antioxidant capacity increased (*P*<0.05) with matrix-based phytase supplementation. Dietary protease supplementation resulted in higher (*P*<0.05) plasma total antioxidant capacity and plasma luteinizing hormone level. Overall, phytase and protease supplementation may benefit laying hens, but responses depend on whether their nutritional matrix values are considered during diet formulation and on their combined use.

## Highlights

• Protease supplementation enhanced egg production, particularly in diets without phytase, and improved internal egg quality parameters such as Haugh unit and yolk color.
• Matrix-based phytase supplementation improved eggshell quality—including thickness, strength, ash content, and calcium composition—while protease in the absence of phytase increased shell phosphorus concentration.
• Phytase (matrix‑based) improved blood antioxidant status by increasing total antioxidant capacity, while protease supplementation enhanced total antioxidant capacity and reduced plasma malondialdehyde concentration in on‑top application, and also increased plasma luteinizing hormone level.

## 1 Introduction

Feed accounts for about 65–70% of poultry production costs, with protein and phosphorus being major contributors (Cai et al., 2024; Pagnussatt et al., 2025). Improving nutrient utilization without affecting performance is essential to reduce costs and environmental impact (Liu et al., 2025). Due to the relatively short digestive tract in poultry, nutrient digestibility may be limited, making exogenous enzyme supplementation an effective strategy to enhance nutrient utilization (Jiang et al., 2020; Poudel et al., 2024).

Although corn and soybean meal contain considerable phosphorus, most is bound as phytic acid and is poorly available to monogastric animals (Ren et al., 2023). Phytate not only limits phosphorus absorption but also impairs nutrient digestibility and performance due to its anti-nutritional effects Saleh et al. (2021). Typical dietary phytate-phosphorus levels range from 2.5 to 4.0 g/kg, while endogenous phytase is insufficient to degrade these complexes (Ren et al., 2023; Sens et al., 2021). Therefore, exogenous phytase is widely used to improve phytate degradation, enhancing nutrient utilization, performance, eggshell quality, and reproductive responses in laying hens (Eltahan et al., 2023; Jlali et al., 2025; Mazhar et al., 2025; Zhai et al., 2022).

A considerable portion of dietary protein (18–20%) escapes digestion by endogenous proteases and is excreted (Cai et al., 2024). This limitation is exacerbated in the late laying stage due to reduced enzyme secretion, leading to impaired digestion and reduced egg production (Chen et al., 2021; Poudel et al., 2024). In addition, soybean meal contains anti-nutritional factors that further hinder nutrient utilization. Therefore, exogenous protease supplementation has been proposed to improve nutrient digestibility and performance, although evidence in laying hens remains limited (Cai et al., 2024; Poudel et al., 2023; Zhu et al., 2022).

The NRC (1994) recommended dietary levels of 3.25% calcium and 0.25–0.45% non‑phytate phosphorus for laying hens. Calcium deficiency can impair bone mineralization, growth, and feed efficiency, whereas excess dietary calcium may also reduce feed efficiency, decrease phytate hydrolysis, limit nutrient availability, and aggravate infections (Paiva et al., 2013; Parsons and Rochell, 2024; Walk and Rao, 2020). Excess dietary calcium can act as an antinutritional factor by forming insoluble calcium–phytate complexes and reducing phosphorus availability, particularly in the small intestine, where the pH is higher than in the upper gastrointestinal tract. High calcium levels may also increase gizzard pH, reducing nutrient absorption and the utilization of phosphorus, energy, and other nutrients (David et al., 2023). Phosphorus is an essential nutrient in poultry nutrition, contributing to eggshell formation, bone development, and mineralization; thus, adequate dietary phosphorus is required to maintain optimal productivity in laying hens (Pagnussatt et al., 2025). However, excessive phosphorus intake may disrupt mineral and vitamin D homeostasis, impair bone metabolism, reduce eggshell quality and feed efficiency, and increase phosphorus excretion, contributing to environmental pollution (Cheng et al., 2025; Mazhar et al., 2025; Pagnussatt et al., 2025; Wang et al., 2021). Therefore, deficiencies, excesses, or imbalances of calcium and phosphorus can disrupt their absorption and homeostasis, leading to reduced performance and poor eggshell quality (Wang et al., 2021). Adequate crude protein is essential to meet amino acid requirements and support optimal performance in laying hens (Mazhar et al., 2025). Although reducing dietary crude protein can lower feed costs and nitrogen excretion, improper balancing may adversely affect performance, health, and egg production (Poudel et al., 2024). Exogenous enzyme supplementation strategies are generally classified as either on-top (without considering the enzyme’s nutritional contribution) or matrix-based (considering the nutrient contributions provided by the enzyme). This distinction is particularly important for calcium, phosphorus, and protein (amino acid) nutrition, as inaccurate estimation of the enzyme’s nutritional contribution may result in nutrient deficiencies or excesses, potentially affecting nutrient balance and overall performance.

Providing diets that closely match nutrient requirements is an effective strategy to improve feed efficiency, reduce production costs, and minimize nitrogen and phosphorus losses to the environment (Macelline et al., 2021). Enzyme supplementation, including phytase and protease, may contribute to this strategy by enhancing nutrient availability and utilization. However, studies investigating the combined supplementation of phytase and protease in late-laying hens, particularly regarding eggshell quality, antioxidant status, gonadotropin concentrations, and ovarian follicular development, are limited. Furthermore, the present study is among the first investigations evaluating the effects of phytase and protease supplementation, with and without considering their nutritional matrix values, on overall performance and physiological responses in laying hens. Therefore, the objective of the present study was to evaluate the effects of phytase and protease supplementation, with and without considering their nutritional matrix values, on productive performance, internal egg quality traits, eggshell characteristics, selected blood metabolites, and ovarian status in late-laying hens.

## 2 Materials and methods

### 2.1 Ethical statement, birds and diets

The experiment was conducted at the Urmia University Research Farm in Iran, and the study protocol was reviewed and approved by the university’s Animal Care and Use Committee. Following a two-week adaptation period, 384 Hy-Line W-80 laying hens at 63 weeks of age were assigned to a 2 × 2 × 2 factorial arrangement consisting of phytase supplementation (0 or 500 FTU/kg), protease supplementation (0 or 5000 U/kg), and diets formulated either with or without consideration of the enzymes’ nutritional matrix values, with six replicates per treatment and eight birds per replicate. The experiment lasted 10 weeks (63 to 73 weeks of age).

The phytase used in the present study was BONFEZYME® (Bonda Faravar Co., Biolouence, Iran), with a guaranteed activity of 10000 U/g. The phytase was supplemented at 50 g per metric ton of feed, resulting in a dietary activity of 500 FTU/kg. The 6-phytase product, derived from a microbial source (*Escherichia coli*), was supplied in a cream-colored powder form. One unit of phytase activity (FTU) was defined as the amount of enzyme required to release 1 μmol of inorganic phosphorus per minute from a 5 mM sodium phytate solution at 37°C and pH 5.5. The protease used in the present study was an alkaline protease supplied by Bonda Faravar Co. (Biolouence), with an activity of 100000 U/g. The protease was supplemented at 50 g per metric ton of feed, providing a dietary activity of 5000 U/kg. The protease of microbial origin (*Bacillus licheniformis*), was provided in a cream-colored powder form. One unit of protease activity was defined as the amount of enzyme capable of releasing 1 μg of tyrosine per minute from casein at 50°C and pH 10.5. The nutritional value of phytase was applied to calcium and phosphorus, while the nutritional value of protease was applied to crude protein, lysine, sulfur‑containing amino acids, and threonine. The activities of phytase and protease in the final diets were not analytically verified (no recovery analysis was conducted), and this should be considered a limitation of the present study.

Prior to the experiment, the corn, soybean meal, and wheat bran used in the formulation of the experimental diets were analyzed, and the values for dry matter, metabolizable energy, starch, crude fiber, acid detergent fiber, neutral detergent fiber, crude protein, ether extract, standardized ileal digestible amino acids, total phosphorus, available phosphorus (total P − phytic P), and phytic phosphorus were estimated using near-infrared reflectance spectroscopy (Paya Amin Mehr, Tehran, Iran). All macronutrients and micronutrients were incorporated into the basal diet in accordance with Hy-Line W-80 nutritional guidelines (Table 1). The dietary Ca and P levels were additionally determined using atomic absorption spectrometry (AOAC Method 927.02, 2000) for calcium and UV spectrophotometry (AOAC Method 965.17, 2000) for phosphorus. The hens were housed in cages providing a floor space of 750 cm² per bird. The experimental period was conducted under a 16-hour light and 8-hour dark cycle, with the house temperature maintained at 18–22°C and relative humidity ranging from 40 to 50%.

**Table 1:** Ingredients and nutrients composition of experimental diets.

***Ingredients***	Nutrient oversupply diet	Nutrient reduced diet	On-top phytase	Matrix-based phytase	On-top protease	Matrix-based protease	On-top phytase + protease	Matrix-based phytase + protease
Corn (ME=3300 kcal/kg)	58.92	60.79	60.26	60.43	60.26	61.27	60.25	60.80
Soy bean meal (CP=41%)	24.62	25.53	25.80	27.11	25.80	25.37	25.79	25.54
Gluten (CP=60%)	2.57	0.00	0.952	0.00	0.951	0.423	0.959	0.00
Wheat bran	0.00	1.57	0.00	0.311	0.00	0.00	0.00	1.53
Soy bean Oil	0.246	0.102	0.179	0.150	0.179	0.139	0.179	0.103
Limestone (Ca=38%)	9.53	10.41	9.96	10.40	9.96	9.96	9.96	10.41
DCP^1^ (Ca=22%, P=18%)	2.68	0.234	1.46	0.235	1.46	1.47	1.46	0.234
Common salt	0.247	0.249	0.248	0.245	0.248	0.248	0.248	0.249
NaHCO_3_	0.150	0.150	0.150	0.150	0.150	0.150	0.150	0.150
Vitamin premix^2^	0.300	0.300	0.300	0.300	0.300	0.300	0.300	0.300
Mineral premix^3^	0.300	0.300	0.300	0.300	0.300	0.300	0.300	0.300
L-Lysine-HCL	0.093	0.034	0.048	0.017	0.048	0.040	0.048	0.033
DL-Methionine	0.261	0.275	0.272	0.285	0.272	0.269	0.272	0.275
L-Threonine	0.074	0.062	0.065	0.063	0.065	0.060	0.065	0.062
Phytase^4^	0.005	0.00	0.005	0.005	0.00	0.00	0.005	0.005
Protease^5^	0.005	0.00	0.00	0.00	0.005	0.005	0.005	0.005
Total	100	100	100	100	100	100	100	100
***Calculated chemical composition (%)***								
ME (kcal/kg)	2600	2600	2600	2600	2600	2600	2600	2600
Crude protein	16.53	15.67	16.10	16.10	16.10	16.10	16.10	16.10
Calcium	4.29	4.09	4.19	4.19	4.19	4.19	4.19	4.19
Available Phosphorous	0.540	0.140	0.340	0.340	0.340	0.340	0.340	0.340
Digestible lysine	0.839	0.801	0.820	0.820	0.820	0.820	0.820	0.820
Digestible Methionine+ cysteine	0.781	0.739	0.760	0.760	0.760	0.760	0.760	0.760
Digestible threonine	0.639	0.601	0.620	0.620	0.620	0.620	0.620	0.620
Linoleic acid	1.52	1.52	1.52	1.52	1.52	1.52	1.52	1.52

^1^DCP: Dicalcium phosphate.

^2^Vitamin premix supplied the following per kilogram of diet. vitamin A (all-trans-retinol), 4400 IU; vitamin D3 (cholecalciferol), 1000 IU; vitamin E (a-tocopherol), 11 IU; vitamin K3 (menadione), 2.33 mg; vitamin B1 (thiamin), 2.97 mg; vitamin B2 (riboflavin), 4.4 mg; vitamin B3 (niacin), 22 mg; vitamin B5 (pantothenic acid), 10 mg; vitamin B6 (pyridoxine), 4.45 mg; vitamin B9 (folic acid), 1.9 mg; vitamin B12 (cyanocobalamin), 0.011 mg; vitamin H2 (biotin), 0.18 mg; choline chloride, 487.5 mg, antioxidant 1.0 mg.

^3^Mineral premix supplied the following per kilogram of diet. Zn (zinc oxide), 75 mg; Mn (manganese oxide), 75; Fe (iron sulfate), 75; Cu (copper sulfate), 5; I (ethylene diamine dihydroiodide), 0.76; Se (Sodium Selenite), 0.1; choline chloride, 474.0

^4^Phytase with 10000 U/g (Bonda Faravar Co., Biolouence, Iran)

^5^Protease with 100000 U/g (Bonda Faravar Co., Biolouence, Iran)

### 2.2 Data collection and sampling

#### 2.2.1 Productive performance

During the experiment, eggs were collected daily, and data on total eggs, egg weight (EW), broken eggs, soft-shelled eggs, and malformed eggs were recorded. Egg collections were conducted at 9:30 a.m. and 3:00 p.m. Weekly feed intake (FI) was also measured throughout the study. Hen-day egg production (EP), EW, egg mass (EM), and feed conversion ratio (FCR) were calculated over the 10-week experimental period. EM was determined by multiplying the EP (%) by EW for each replicate, while FCR was calculated as the grams of FI per gram of EM produced.

#### 2.2.2 Internal egg quality assessment

To evaluate the internal quality of eggs, two eggs per replicate were collected over three consecutive days (36 eggs per treatment) during the last week of the experiment. The assessment included shape index (SI), albumen ratio (AR), yolk ratio (YR), Albumen height (AH), Haugh units (HU), and yolk color (YC). Each egg was weighed on an electronic scale (Jy602; Shanghai Puchun Measure Instrument) with 0.01 g precision prior to breaking. Egg length (longitudinal axis) and width (equatorial axis) were measured using a digital sliding caliper (Baking Win) with 0.01 mm precision. The SI was then calculated as follows: SI (%) = (egg width in mm/egg length in mm) ×100. To determine egg component weights, each yolk was individually weighed using a laboratory balance with 0.01 g precision. Albumen weight was obtained by subtracting the combined yolk and shell weights from the total egg weight. The proportions of yolk and albumen were calculated using the formula: component ratio (%) = (component weight / egg weight) × 100. The AH was measured by placing the tip of the Haugh meter ruler 10 mm from the yolk margin, and the HU was then calculated using the equation HU = 100 × log (AH– 1.7 × EW^0.37^ + 7.57). The YC was determined using a DSM Yolk Color Fan (Switzerland), which classifies pigmentation on a graded scale ranging from 1 (light yellow) to 15 (intense red–orange).

#### 2.2.3 Egg shell status

Shell weight was recorded after washing and drying the shells at 60°C for 48 h. The shell ratio (SR) was calculated as (egg shell weight / egg weight) × 100. The egg shell thickness (EST) was measured by marking and cutting a 1 cm² section of the shell at three locations, the equator, the narrow end, and the wide end. The thickness of each shell segment (excluding the membrane) was determined using a micrometer (0.01 mm; YP001, Mitutoyo), and the mean of the three measurements was taken as the EST. The egg shell strength (ESS), expressed in kg/cm², was measured using an egg-breaking strength apparatus (Ogawa Seiki Co. Ltd., Tokyo, Japan), in which each egg was positioned vertically and compressed until shell rupture; the peak force required to break the shell was recorded. To determine the Ca and P concentrations in egg shells, samples were dried, finely ground, and subjected to acid digestion using sulfuric acid. Approximately 0.1 g of the ground sample was ashed overnight at 600 °C in a muffle furnace, then allowed to cool to room temperature. The ash was then digested with 10 ml of 7.4 M sulfuric acid at its boiling point for 1 hour. After cooling, the digested material was filtered through Whatman No. 541 filter paper into a 100 ml volumetric flask, brought to volume with distilled water, and subsequently analyzed for Ca and P. The Ca was quantified using atomic absorption spectrometry according to AOAC 2000, Method 927.02, and total phosphorus was measured by UV spectrophotometry following AOAC 2000, Method 965.17.

#### 2.2.4 Plasma biochemical metabolites

At the end of the ten-week experimental period, 3 mL of blood were collected from two hens per cage (12 hens per treatment) via wing-vein venipuncture. Blood samples were drawn into heparinized glass tubes and centrifuged at 3500 × g for 10 minutes at 4°C to obtain plasma, which was then stored at –20°C until analysis. Plasma concentrations of glucose, albumin, total protein, cholesterol, triglycerides, high-density lipoprotein, uric acid, Ca, and P were measured using commercial enzymatic kits (Pars Azmoon Inc., Tehran, Iran) with a plasma autoanalyzer (Abbott Laboratories, Illinois, USA). Additionally, total antioxidant capacity (TAC) and malondialdehyde (MDA) levels were determined colorimetrically using ZellBio kits (ZellBio GmbH, Ulm, Germany) according to the manufacturer’s instructions.

#### 2.2.5 Plasma gonadotropins and ovarian follicle assessment

The EMP-830Vet diagnostic device (Operation Manual) was used to non-invasively determine the number and diameter of ovarian follicles. Images were obtained using a radial panoramic endovaginal transducer (5 and 7.5 MHz) with a diameter of 20 mm. Each hen was restrained by placing it on its breast while an assistant held its legs to keep it immobile. To ensure uninterrupted transmission of sound waves, ultrasound gel was applied to the surface of the transducer. The transducer was inserted 3–5 cm into the hen’s cloaca at an approximate 30° angle relative to the dorsal wall. To standardize the transducer position, the orientation marker on the probe was consistently aligned with the hen’s dorsal midline. The presence of an egg in the vagina could interfere with ultrasound examinations, as it prevented proper positioning of the transducer. To reduce the likelihood of such an occurrence, ultrasound examinations were performed after 14:00. Ultrasound examinations were performed in brightness mode, and the area surrounding the ovary was scanned. To achieve a complete scan of the ovary, once a follicle was identified, the transducer was moved to cover the entire ovarian region. Plasma concentrations of follicle‑stimulating hormone (FSH) and luteinizing hormone (LH) were also determined using an enzyme-linked immunosorbent assay kit.

#### 2.2.6 Economic analysis

This economic analysis was based on local feed prices and is presented as an example rather than a general recommendation. Results may vary across regions; therefore, calculations should be performed using current regional prices. Labor and capital costs were assumed to be constant across treatments, as all groups were managed under identical conditions, including feeding strategies, housing systems, and equipment. These costs were therefore excluded from the analysis. The evaluation included feed cost, egg revenue, net profit, and the cost–benefit ratio. Feed cost was calculated as FI (kg) × feed price per kilogram. Egg revenue was calculated as EM (kg) × egg price per kilogram. Net profit was defined as egg revenue minus feed cost. The cost–benefit ratio was calculated as egg revenue divided by feed cost. Feed cost per kilogram of egg mass was calculated by multiplying the feed-to-egg mass ratio by the feed price per kilogram of diet.

### 2.3 Statistical analyses

The data obtained in this study were analyzed using a 2×2×2 factorial arrangement within a completely randomized design. The experimental factors consisted of varying levels of phytase enzyme supplementation (0 and 500 FTU/kg), protease enzyme supplementation (0 and 5000 U/kg), and the inclusion or exclusion of the nutritional value of the enzymes, with six replicates assigned to each treatment. The statistical model for productive performance traits data was represented as: Y_ijkl_ = *μ* + A_i_ + B_j_ + C_l_ + AB_ij_ + AC_il_ + BC_jl_ + ABC_ijl_ + e_ijlk_. For other variables, the model applied was: Y_ijlkm_ = *μ* + A_i_ + B_j_ + C_l_ + AB_ij_ + AC_il_ + BC_jl_ + ABC_ijl_ + e_ijlk_ + ε_ijlkm_. The components of the models are defined as follows: $Y_{ijkl}$and $Y_{ijklm}$represent the observed traits, μis the overall mean, $A_{i}$, $B_{j}$, and $C_{l}\;$denote the main effects of phytase supplementation, protease supplementation, and inclusion or exclusion of the nutritional value of the enzymes, respectively, and $AB_{ij}$, $AC_{il}$, $BC_{jl}$, and $ABC_{ijl}$represent their two- and three-way interactions. The term $e_{ijkl}$accounts for the experimental error, and $\varepsilon_{ijklm}$represents the subsampling error. For productive performance traits, the cage (replicate) was considered the experimental unit. For internal egg quality traits, blood parameters, eggshell characteristics, plasma gonadotropins, and ovarian follicle assessments, measurements obtained from individual birds or eggs within each replicate were treated as subsamples, with the cage considered the experimental unit. Prior to statistical analysis, the normality of both the raw data and the residuals were evaluated using the Shapiro–Wilk, Kolmogorov–Smirnov, Cramér–von Mises, and Anderson–Darling tests within the UNIVARIATE procedure of SAS. Homogeneity of variances was also assessed using Levene’s test. Outliers were identified and excluded when their values exceeded the mean ± three standard deviations. Treatment means were compared using the PDIFF option in SAS, with Tukey's adjustment applied when significant effects were identified. Statistical significance was set at P < 0.05. Simple effect analysis and mean comparisons were also performed to interpret significant interaction effects when observed.

## 3 Result

### 3.1 Productive performance

As shown in Table 2, the main effects of enzyme supplementation, with or without considering the enzyme nutritional matrix, and their interaction did not significantly (*P* > 0.05) influence EW, EM, or FCR. On-top enzymes supplementation increased (*P* = 0.032) EP by 2.74% compared with the matrix-based diets. Furthermore, EP was significantly (*P* = 0.009) affected by the interaction between phytase and protease supplementation. The inclusion of protease in diets without phytase increased EP by 3.73% compared with diets without enzyme supplementation, whereas this effect was not observed when both enzymes were supplemented together.

**Table 2:** Effects of phytase and protease supplementation, with and without considering their nutritional matrix values, on the productive performance of laying hens during 63–73 weeks of age.

*Factorial analysis*	Egg weight (g)	Egg production (%)	Egg mass (g)	FCR
Phy* (FTU/kg)				
0	55.19	90.36	49.96	2.11
500	55.17	89.50	49.50	2.13
*SEM*	0.633	0.777	0.957	0.044
**Pro^&^** (U/kg)				
0	54.53	89.79	49.05	2.14
5000	55.82	90.08	50.40	2.10
*SEM*	0.633	0.777	0.957	0.044
**NMV ^#^**				
On top base	55.66	91.15^a^	50.83	2.08
Matrix based	54.69	88.72^b^	48.63	2.16
*SEM*	0.633	0.777	0.957	0.044
**Phy** (FTU/kg) **× Pro** (U/kg)				
0 × 0	54.14	88.71^b^	48.10	2.18
0 × 5000	56.23	92.02^a^	51.80	2.05
500 × 0	54.92	90.86^ab^	50.00	2.10
500 × 5000	55.41	88.16^b^	49.00	2.15
*SEM*	0.896	1.10	1.35	0.062
**Phy** (FTU/kg) **× NMV**				
0 × On top base	56.00	91.97	51.54	2.06
0 × Matrix base	54.38	88.77	48.35	2.23
500 × On top	55.32	90.34	50.10	2.10
500 × Matrix base	55.01	88.66	48.90	2.09
*SEM*	0.896	1.10	1.35	0.062
**Pro** (U/kg) **× NMV**				
0 × On top base	55.63	91.57	51.02	2.07
0 × Matrix base	53.43	88.00	47.08	2.24
5000 × On top	55.68	90.74	50.63	2.10
5000 × Matrix base	55.96	89.43	50.17	2.11
*SEM*	0.896	1.10	1.35	0.062
**Phy** (FTU/kg) **× Prot** (U/kg) **× NMV**				
0 × 0 × On top base	55.71	90.74	50.63	2.07
0 × 0 × Matrix base	52.58	86.69	45.59	2.28
0 × 5000 × On top base	56.28	93.18	52.46	2.04
0 × 5000 × Matrix base	56.18	90.85	51.13	2.05
500 × 0 × On top base	55.56	92.41	51.42	2.04
500 × 0 × Matrix base	54.28	89.30	48.58	2.17
500 × 5000 × On top base	55.08	88.28	48.79	2.16
500 × 5000 × Matrix base	55.74	88.03	49.21	2.14
*SEM*	1.26	1.55	1.91	0.087
				***P-value***
Phy	0.981	0.441	0.741	0.809
Pro	0.158	0.784	0.325	0.496
NMV	0.288	0.032	0.112	0.195
Phy × Pro	0.379	0.009	0.090	0.161
Phy × NMV	0.471	0.495	0.470	0.659
Pro × NMV	0.172	0.304	0.205	0.154
Phy × Pro × NMV	0.765	0.797	0.940	0.799

Means in the same column with different superscripts differ significantly (*P* < 0.05). Each mean represents values from 6 replicates. SEM: standard error of means.

*Phy: phytase, ^&^Pro: protease, ^#^NMV: nutritional matrix value. FCR: feed conversion ratio.

### 3.2 Internal egg quality assessment

Table 3 presents the effects of phytase and protease supplementation, with or without accounting for their nutritional contributions, and their interactions on internal egg quality traits. No significant (*P* > 0.05) effects of phytase or protease supplementation, with or without consideration of their nutritional values, or their interactions were observed on YR, AR, and SI. In contrast, protease supplementation significantly increased the Haugh unit (*P* = 0.026; by 2.88%) and yolk color score (*P* = 0.043; by 5.00%) compared with diets without protease supplementation.

**Table 3:** Effects of phytase and protease supplementation, with and without considering their nutritional matrix values, on internal egg quality traits of laying hens at 73 weeks of age.

*Factorial analysis*	Yolk (%)	Albumen (%)	Yolk color	Haugh unit	Shape index
Phy* (FTU/kg)					
0	28.30	61.70	3.70	79.39	74.50
500	28.24	61.68	3.68	79.36	74.95
*SEM*	0.172	0.180	0.060	0.700	0.277
**Pro^&^** (U/kg)					
0	28.37	61.62	3.60^b^	78.25^b^	74.58
5000	28.19	61.76	3.78^a^	80.50^a^	74.89
*SEM*	0.172	0.180	0.060	0.700	0.277
**NMV ^#^**					
On top base	28.32	61.70	3.71	79.25	74.70
Matrix based	28.22	61.69	3.67	79.50	74.77
*SEM*	0.172	0.180	0.060	0.700	0.277
**Phy** (FTU/kg) × **Pro** (U/kg)					
0 × 0	28.37	61.66	3.57	79.19	74.14
0 × 5000	28.24	61.76	3.84	79.59	74.88
500 × 0	28.37	61.60	3.63	77.31	74.99
500 × 5000	28.12	61.76	3.72	81.40	74.90
*SEM*	0.243	0.255	0.085	0.990	0.391
**Phy** (FTU/kg) × **NMV**					
0 × On top base	28.24	61.85	3.77	78.53	74.50
0 × Matrix base	28.36	61.57	3.63	80.25	74.51
500 × On top	28.41	61.55	3.65	79.99	74.89
500 × Matrix base	28.09	61.81	3.70	78.73	75.00
*SEM*	0.243	0.255	0.085	0.990	0.391
**Pro** (U/kg) × **NMV**					
0 × On top base	28.31	61.80	3.64	78.52	74.18
0 × Matrix base	28.41	61.45	3.57	77.99	74.97
5000 × On top	28.34	61.60	3.78	79.98	75.20
5000 × Matrix base	28.02	61.92	3.77	81.00	74.57
*SEM*	0.243	0.255	0.085	0.990	0.391
**Phy** (FTU/kg) × **Prot** (U/kg) × **NMV**					
0 × 0 × On top base	28.15	62.03	3.59	78.40	73.74
0 × 0 × Matrix base	28.58	61.27	3.55	79.99	74.57
0 × 5000 × On top base	28.34	61.65	3.96	78.65	75.26
0 × 5000 × Matrix base	28.15	61.86	3.70	80.52	74.47
500 × 0 × On top base	28.48	61.55	3.69	78.66	74.63
500 × 0 × Matrix base	28.25	61.64	3.58	75.98	75.35
500 × 5000 × On top base	28.34	61.54	3.60	81.30	75.14
500 × 5000 × Matrix base	27.90	61.99	3.84	81.49	74.65
*SEM*	0.344	0.360	0.121	1.40	0.553
					***P-value***
Phy	0.807	0.920	0.775	0.977	0.269
Pro	0.468	0.614	0.043	0.026	0.423
NMV	0.670	0.974	0.604	0.805	0.866
Phy × Pro	0.799	0.904	0.318	0.065	0.296
Phy × NMV	0.360	0.290	0.240	0.135	0.904
Pro × NMV	0.390	0.190	0.708	0.428	0.071
Phy × Pro × NMV	0.698	0.555	0.109	0.515	0.806

Means in the same column with different superscripts differ significantly (*P* < 0.05). Each mean represents values from 6 replicates (n=36). SEM: standard error of means.

*Phy: phytase, ^&^Pro: protease, ^#^NMV: nutritional matrix value.

### 3.3 Egg shell status

Table 4 presents the effects of phytase and protease supplementation, with or without accounting for their nutritional contributions, as well as their interactions, on eggshell characteristics. Phytase or protease supplementation, with or without consideration of their nutritional values and their interactions, had no significant (*P* > 0.05) effect on SR. The EST was significantly influenced (P = 0.001) by the interaction among the studied factors. Matrix-based phytase supplementation resulted in greater EST compared with the nutrient-reduced diet (by 7.28%), protease-supplemented diets (by 9.07% in the on-top and 7.53% in the matrix-based treatments), and the on-top phytase supplementation diet (by 5.79%). However, phytase supplementation in an on-top manner did not result in an increase in EST compared with the nutrient-reduced diet or diets supplemented with protease alone. The ESS was affected (*P* = 0.039) by the interaction between phytase supplementation and matrix consideration, with higher values observed in matrix-based phytase treatments compared with phytase-free diets (by 13.93% in oversupplied and 10.18% in nutrient-reduced conditions) and on-top phytase diets (by 13.58%). However, no improvement in ESS was observed with on-top phytase supplementation. A significant (*P* = 0.040) three-way interaction among the studied factors affected eggshell calcium content. Matrix-based phytase supplementation increased eggshell calcium content compared with enzyme-free diets (by 3.66% in the nutrient-oversupplied and 5.05% in the nutrient-reduced diets), protease-supplemented diets (by 3.68% in the on-top and 4.28% in the matrix-based diets), and on-top phytase supplementation (by 4.86%). However, this difference was not observed in diet supplemented with phytase in an on-top manner or in combination with protease compared with the other experimental treatments.

**Table 4:** Effects of phytase and protease supplementation, with and without considering their nutritional matrix v alues, on egg shell status of laying hens at 73 weeks of age.

*Factorial analysis*	Shell (%)	EST (mm)	ESS (kg/cm^2^)	Ash (%)	Ca (%)	P (%)
Phy* (FTU/kg)						
0	9.99	0.428	3.30	90.94	34.37	0.310
500	10.08	0.444	3.46	91.93	34.91	0.317
*SEM*	0.048	0.002	0.053	0.284	0.203	0.005
Pro^&^ (U/kg)						
0	10.01	0.439	3.36	91.22	34.64	0.307
5000	10.06	0.433	3.40	91.66	34.63	0.320
*SEM*	0.048	0.002	0.053	0.284	0.203	0.005
NMV ^#^						
On top base	9.98	0.435	3.24	91.50	34.57	0.315
Matrix based	10.09	0.438	3.51	91.37	34.71	0.312
*SEM*	0.048	0.002	0.053	0.284	0.203	0.005
Phy (FTU/kg) × Pro (U/kg)						
0 × 0	9.98	0.435	3.26	90.58	34.31	0.297^b^
0 × 5000	9.99	0.422	3.32	91.31	34.43	0.324^a^
500 × 0	10.04	0.444	3.45	91.86	34.98	0.317^ab^
500 × 5000	10.12	0.443	3.48	92.00	34.85	0.316^ab^
*SEM*	0.068	0.003	0.076	0.401	0.287	0.007
Phy (FTU/kg) × NMV						
0 × On top base	10.91	0.431	3.23^b^	91.44^ab^	34.54	0.323^ab^
0 × Matrix base	10.07	0.425	3.34^b^	90.44^b^	34.21	0.298^b^
500 × On top	10.04	0.438	3.24^b^	91.56^ab^	34.60	0.307^ab^
500 × Matrix base	10.11	0.450	3.68^a^	92.30^a^	35.20	0.327^a^
*SEM*	0.068	0.003	0.076	0.401	0.287	0.007
Pro (U/kg) × NMV						
0 × On top base	9.89	0.438	3.17	90.96	34.34	0.311
0 × Matrix base	10.13	0.441	3.55	91.48	34.95	0.303
5000 × On top	10.06	0.431	3.32	92.04	34.80	0.319
5000 × Matrix base	10.05	0.434	3.48	91.27	34.48	0.322
*SEM*	0.068	0.003	0.076	0.401	0.287	0.007
Phy (FTU/kg) × Prot (U/kg) × NMV						
0 × 0 × On top base	9.81	0.444^ab^	3.22	90.78	34.54^b^	0.312
0 × 0 × Matrix base	10.15	0.426^bc^	3.31	90.38	34.08^b^	0.281
0 × 5000 × On top base	10.01	0.419^c^	3.25	92.11	34.53^b^	0.334
0 × 5000 × Matrix base	9.99	0.425^bc^	3.38	90.51	34.33^b^	0.315
500 × 0 × On top base	9.97	0.432^bc^	3.11	91.16	34.14^b^	0.310
500 × 0 × Matrix base	10.10	0.457^a^	3.79	92.58	35.80^a^	0.326
500 × 5000 × On top base	10.12	0.443^ab^	3.38	91.97	35.07^ab^	0.304
500 × 5000 × Matrix base	10.11	0.444^ab^	3.57	92.03	34.62^ab^	0.329
*SEM*	0.096	0.004	0.107	0.568	0.406	0.010
						*P-value*
Phy	0.214	<0.0001	0.030	0.015	0.066	0.358
Pro	0.476	0.053	0.635	0.279	0.988	0.064
NMV	0.101	0.386	0.001	0.755	0.635	0.718
Phy × Pro	0.643	0.073	0.847	0.461	0.666	0.038
Phy × NMV	0.486	0.011	0.039	0.032	0.110	0.001
Pro × NMV	0.068	0.967	0.130	0.114	0.113	0.457
Phy × Pro × NMV	0.411	0.001	0.088	0.920	0.040	0.910

Means in the same column with different superscripts differ significantly (*P* < 0.05). Each mean represents values from 6 replicates (n=36). SEM: standard error of means.

*Phy: phytase, ^&^Pro: protease, ^#^NMV: nutritional matrix value. EST: egg shell thickness, ESS: egg shell strength, Ca: calcium, P: phosphorous

Eggshell phosphorus content was affected (*P* = 0.038) by the interaction between phytase and protease supplementation, and both eggshell phosphorus (*P* = 0.001) and eggshell ash (*P* = 0.032) contents were influenced by the interaction between phytase supplementation and matrix consideration. Supplementation with protease alone increased eggshell phosphorus content by 9.09% compared with diets without enzyme supplementation, whereas this effect was not observed in diets supplemented with both phytase and protease. Furthermore, eggshell phosphorus and ash contents were higher in the matrix-based phytase supplementation diet than in the nutrient-reduced diet (by 9.73% and 2.06%, respectively), whereas no differences were observed between the on-top phytase supplementation diet and the other experimental groups.

### 3.4 Plasma biochemical metabolites

Table 5 illustrates the effects of phytase and protease supplementation, with or without accounting for their nutritional values, as well as their interactions, on plasma concentrations of selected blood metabolites. Plasma concentrations of albumin, glucose, HDL, cholesterol, and uric acid were not influenced (*P* > 0.05) by enzymes supplementation, consideration of enzymes matrix values, or their interactions. However, plasma total protein levels were significantly increased by phytase (*P* = 0.042) and protease (*P* = 0.010) supplementation, by 3.63% and 4.64%, respectively, compared with diets without enzymes supplementation. Protease supplementation also led to significant increases in plasma calcium (by 5.99%; *P* < 0.0001) and phosphorus concentrations (by 5%; *P* = 0.031) compared with diets lacking enzyme supplementation. Plasma calcium (*P* = 0.021) and phosphorus (*P* = 0.043) were also affected by the interaction between phytase supplementation and matrix consideration. On-top phytase supplementation increased plasma calcium compared with phytase-free diets (by 9.12% and 17.7% in oversupplied and nutrient-reduced diets, respectively), whereas matrix-based phytase had no effect. Conversely, matrix-based phytase supplementation increased plasma phosphorus compared with phytase-free diets (by 9.07% and 11.93% in oversupplied and nutrient-reduced diets, respectively), whereas no differences were observed between on-top phytase supplementation and phytase-free diets. Protease supplementation increased TAC by 7.10% (*P* = 0.011) compared with enzyme-free diets. Plasma TAC was affected (*P* = 0.006) by the phytase × matrix value consideration interaction. Phytase supplementation, whether applied in an on-top or matrix-based manner, increased plasma TAC concentration by 11.56% and 22.45%, respectively, compared with the nutrient-reduced diet. However, only matrix-based phytase increased plasma TAC by 17.26% compared with the nutrient-oversupplied diet. Simple effects analysis showed that, without phytase supplementation, applying matrix values increased (*P* = 0.001) MDA by 9.32% compared with the nutrient-oversupplied diet, but had no effect when phytase was included. Furthermore, MDA concentration was affected (*P* = 0.032) by the interaction between protease supplementation and matrix consideration. In protease-containing diets, on-top protease supplementation reduced MDA concentration by 11.18% compared with the oversupplied diet.

**Table 5:** Effects of phytase and protease supplementation, with and without considering their nutritional matrix values, on blood metabolites in laying hens at 510 d.

*Factorial analysis*	Glu (mg/dl)	TP (g/dl)	ALB (g/dl)	Chol (mg/dl)	HDL (mg/dl)	Uric acid (mg/dl)	Ca (mg/dl)	P (mg/dl)	TAC (mmol/ml)	MDA (nmol/ml)
Phy* (FTU/kg)										
0	245.9	6.06^b^	3.74	169.7	71.39	6.35	12.68	7.72	3.00	1.69
500	254.9	6.28^a^	3.89	172.5	73.04	6.14	13.52	8.27	3.43	1.55
*SEM*	4.32	0.077	0.057	1.16	0.849	0.119	0.110	0.127	0.059	0.027
Pro^&^ (U/kg)										
0	252.3	6.03^b^	3.76	169.8	71.46	6.40	12.76^b^	7.80^b^	3.10^b^	1.67
5000	248.4	6.31^a^	3.88	172.3	72.97	6.10	13.44^a^	8.19^a^	3.32^a^	1.57
*SEM*	4.32	0.077	0.057	1.16	0.849	0.119	0.110	0.127	0.059	0.027
NMV ^#^										
On top base	249.4	6.21	3.82	171.1	71.39	6.40	13.77^a^	7.91	3.17	1.61
Matrix based	251.3	6.13	3.80	170.9	73.04	6.10	12.43^b^	8.08	3.27	1.63
SEM	4.32	0.077	0.057	1.16	0.849	0.119	0.110	0.127	0.059	0.027
Phy (FTU/kg) × Pro (U/kg)										
0 × 0	248.2	5.90	3.72	168.3	71.61	6.44	12.37	7.50	2.89	1.76
0 × 5000	243.5	6.23	3.76	170.9	71.17	6.25	12.99	7.96	3.11	1.61
500 × 0	256.5	6.17	3.78	171.1	71.31	6.36	13.13	8.10	3.33	1.57
500 × 5000	253.3	6.40	3.99	173.9	74.77	5.94	13.90	8.42	3.52	1.52
*SEM*	6.12	0.109	0.080	1.63	1.20	0.169	0.157	0.178	0.083	0.038
Phy (FTU/kg) × NMV										
0 × On top base	243.9	6.09	3.81	170.0	70.99	6.46	13.16^b^	7.83^b^	3.07^bc^	1.61^b^
0 × Matrix base	247.9	6.03	3.67	169.1	71.80	6.24	12.20^c^	7.63^b^	2.94^c^	1.76^a^
500 × On top	255.0	6.34	3.83	172.2	71.79	6.35	14.36^a^	7.99^ab^	3.28^b^	1.60^b^
500 × Matrix base	254.9	6.22	3.92	172.7	74.29	5.95	12.68^bc^	8.54^a^	3.60^a^	1.49^b^
*SEM*	6.12	0.109	0.080	1.63	1.20	0.169	0.157	0.179	0.083	0.038
Pro (U/kg) × NMV										
0 × On top base	250.7	6.02	3.79	169.1	70.80	6.43	13.47	7.72	3.03	1.70^a^
0 × Matrix base	254.0	6.04	3.72	170.3	72.14	6.37	12.04	7.87	3.19	1.63^ab^
5000 × On top	248.1	6.41	3.87	173.2	71.99	6.37	14.06	8.09	3.30	1.51^b^
5000 × Matrix base	248.8	6.21	3.88	171.5	73.95	5.82	12.83	8.30	3.34	1.62^ab^
*SEM*	6.12	0.109	0.080	1.63	1.20	0.169	0.157	0.179	0.083	0.038
Phy (FTU/kg) × Prot (U/kg) × NMV										
0 × 0 × On top base	248.9	5.83	3.80	169.5	72.09	6.40	12.87	7.57	2.89	1.71
0 × 0 × Matrix base	247.5	5.95	3.66	167.2	71.14	6.49	11.89	7.43	2.89	1.82
0 × 5000 × On top base	238.9	6.34	3.82	170.7	69.90	6.52	13.45	8.09	3.25	1.51
0 × 5000 × Matrix base	248.3	6.12	3.69	171.0	72.43	5.99	12.53	7.83	2.99	1.71
500 × 0 × On top base	252.5	6.20	3.77	168.8	69.49	6.47	14.06	7.90	3.18	1.68
500 × 0 × Matrix base	260.5	6.13	3.79	173.5	73.14	6.26	12.21	8.30	3.48	1.45
500 × 5000 × On top base	257.5	6.49	3.91	175.8	74.07	6.22	14.68	8.09	3.35	1.52
500 × 5000 × Matrix base	249.1	6.31	4.06	172.0	75.46	5.64	13.12	8.76	3.70	1.53
*SEM*	8.66	0.154	0.113	2.31	1.70	0.238	0.221	0.252	0.116	0.053
										*P-value*
Phy	0.145	0.042	0.080	0.083	0.173	0.235	<.0001	0.003	<.0001	0.001
Pro	0.523	0.010	0.144	0.112	0.214	0.069	<.0001	0.031	0.011	0.011
NMV	0.750	0.426	0.704	0.899	0.172	0.070	<.0001	0.338	0.251	0.600
Phy × Pro	0.902	0.622	0.277	0.939	0.108	0.466	0.644	0.701	0.804	0.148
Phy × NMV	0.729	0.769	0.154	0.667	0.477	0.595	0.021	0.043	0.006	0.001
Pro × NMV	0.822	0.310	0.690	0.376	0.801	0.132	0.575	0.849	0.515	0.032
Phy × Pro × NMV	0.270	0.599	0.686	0.091	0.234	0.707	0.689	0.594	0.323	0.382

Means in the same column with different superscripts differ significantly (*P* < 0.05). Each mean represents values from 6 replicates (n=12). SEM: standard error of means.

*Phy: phytase, &Pro: protease, #NMV: nutritional matrix value. Glu: glucose, TP: total protein, ALB: albumin, Chol: cholesterol, HDL: high density lipoprotein, Ca: calcium, P: phosphorous, TAC: total antioxidant capacity, MDA: malondialdehyde.

### 3.5 Plasma gonadotropins and ovarian follicle assessment

The effects of phytase and protease supplementation, with or without consideration of their nutritional matrix values, and their interactions on plasma concentrations of FSH and LH, as well as ovarian follicular status, are presented in Table 6. Plasma FSH concentration and the diameters of F3, F4, and F5 follicles were not affected (*P* > 0.05) by phytase or protease supplementation, matrix consideration, or their interactions. However, protease supplementation increased plasma LH concentration (*P* = 0.025) and the diameters of F1 (*P* = 0.049) and F2 (*P* = 0.019) follicles by 5.51%, 6.38%, and 5.44%, respectively.

**Table 6:** Effects of phytase and protease supplementation, with and without considering their nutritional matrix values, on plasma gonadotropins and ovarian follicle assessment in laying hens at 511 d.

*Factorial analysis*	F1 (mm)	F2 (mm)	F3 (mm)	F4 (mm)	F5 (mm)	FSH (mIU/ml)	LH (mIU/ml)
Phy* (FTU/kg)							
0	38.28	31.85	24.68	21.14	17.17	41.04	28.15
500	38.33	31.95	25.65	21.41	17.78	42.39	28.12
*SEM*	0.798	0.489	0.461	0.190	0.240	0.533	0.463
Pro^&^ (U/kg)							
0	37.12^b^	31.06^b^	24.94	21.12	17.41	41.03	27.38^b^
5000	39.49^a^	32.75^a^	25.39	21.43	17.54	42.41	28.89^a^
*SEM*	0.798	0.489	0.461	0.190	0.240	0.533	0.463
NMV ^#^							
On top base	37.80	31.87	25.51	21.26	17.35	42.03	27.61
Matrix based	38.81	31.94	24.82	21.29	17.60	41.41	28.66
SEM	0.798	0.489	0.461	0.190	0.240	0.533	0.463
Phy (FTU/kg) × Pro (U/kg)							
0 × 0	37.89	30.73	25.06	20.92	17.28	40.27	27.33
0 × 5000	38.67	32.98	24.30	21.35	17.06	41.82	28.97
500 × 0	36.35	31.39	24.82	21.32	17.53	41.79	27.43
500 × 5000	40.31	32.52	26.48	21.51	18.03	43.00	28.82
*SEM*	1.12	0.692	0.653	0.268	0.340	0.754	0.655
Phy (FTU/kg) × NMV							
0 × On top base	37.13	31.72	24.65	21.29	17.13	41.65	27.60
0 × Matrix base	39.42	31.99	24.71	20.99	17.21	40.43	28.70
500 × On top	38.47	32.02	26.37	21.23	17.58	42.41	27.62
500 × Matrix base	38.20	31.89	24.94	21.60	17.98	42.38	28.63
*SEM*	1.12	0.692	0.653	0.268	0.340	0.754	0.655
Pro (U/kg) × NMV							
0 × On top base	37.32	31.51	25.80	21.07	17.37	41.06	26.93
0 × Matrix base	36.92	30.62	24.08	21.18	17.44	41.00	27.82
5000 × On top	38.28	32.23	25.22	21.46	17.33	43.01	28.28
5000 × Matrix base	40.70	33.26	25.56	21.40	17.75	41.81	29.50
*SEM*	1.12	0.692	0.653	0.268	0.340	0.754	0.655
Phy (FTU/kg) × Prot (U/kg) × NMV							
0 × 0 × On top base	37.70	30.91	25.63	21.30	17.45	40.48	26.82
0 × 0 × Matrix base	38.07	30.56	24.49	20.55	17.11	40.06	27.83
0 × 5000 × On top base	36.56	32.53	23.68	21.28	16.81	42.83	28.37
0 × 5000 × Matrix base	40.78	33.42	24.92	21.43	17.31	40.81	29.57
500 × 0 × On top base	36.94	32.11	25.97	20.84	17.29	41.63	27.04
500 × 0 × Matrix base	35.77	30.67	23.68	21.81	17.77	41.94	27.82
500 × 5000 × On top base	40.00	31.93	26.76	21.63	17.86	43.19	28.20
500 × 5000 × Matrix base	40.62	33.10	26.20	21.38	18.20	42.81	29.44
*SEM*	1.59	0.979	0.923	0.380	0.481	1.06	0.926
							*P-value*
Phy	0.961	0.887	0.144	0.310	0.080	0.081	0.968
Pro	0.042	0.019	0.496	0.261	0.686	0.074	0.025
NMV	0.376	0.922	0.298	0.911	0.474	0.409	0.115
Phy × Pro	0.167	0.424	0.072	0.648	0.298	0.825	0.847
Phy × NM	0.262	0.773	0.263	0.255	0.635	0.438	0.945
Pro × NMV	0.219	0.173	0.124	0.765	0.613	0.451	0.803
Phy × Pro × NMV	0.649	0.625	0.804	0.057	0.475	0.762	0.914

Means in the same column with different superscripts differ significantly (*P* < 0.05). Each mean represents values from 6 replicates (n=12). SEM: standard error of means. *Phy: phytase, ^&^Pro: protease, ^#^NMV: nutritional matrix value. F1–F5 (mm): hierarchical preovulatory follicles arranged from larger to smaller. FSH: follicle‑stimulating hormone, LH: luteinizing hormone.

### 3.6 Economic analysis

Table 7 presents the effects of phytase and protease supplementation, with or without inclusion of matrix values, and their interaction on key economic indicators (feed cost, egg revenue, net profit, cost–benefit ratio, and feed cost per kilogram of EM). Feed cost was influenced (*P* < 0.0001) by the phytase × protease × matrix values interaction. The nutrient oversupply diet exhibited the highest feed cost, with a significant difference compared to the other treatments. Matrix-based phytase supplementation reduced feed cost compared with on-top phytase supplementation, with a feed cost comparable to that of the nutrient-reduced diet. No difference in feed cost was observed between matrix-based and on-top protease supplementation. However, combined supplementation of phytase and protease using matrix values resulted in a lower feed cost compared with diets supplemented with protease alone, either in an on-top or matrix-based manner, and was comparable to the nutrient-reduced diet. Egg revenue, net profit, cost–benefit ratio, and feed cost per kilogram of EM were not affected (*P* > 0.05) by phytase and protease supplementation, matrix value consideration, or their interactions.

**Table 7:** Effects of phytase and protease supplementation, with and without considering their nutritional matrix values, on economics evaluation of laying hens during 63–73 weeks of age.

*Factorial analysis*	Feed cost ($/hen/70 d)	Egg revenue ($/hen/70 d)	Net profit ($/hen/70 d)	Cost-benefit Ratio	FCE^@^ ($)
Phy* (FTU/kg)					
0	2.22	4.21	1.98	1.90	0.643
500	2.17	4.19	2.03	1.94	0.632
*SEM*	0.004	0.084	0.085	0.039	0.013
Pro^&^ (U/kg)					
0	2.20	4.15	1.96	1.89	0.645
5000	2.19	4.25	2.06	1.94	0.630
*SEM*	0.004	0.084	0.085	0.039	0.013
NMV ^#^					
On top base	2.27	4.28	2.01	1.89	0.647
Matrix based	2.12	4.12	2.00	1.95	0.628
*SEM*	0.004	0.084	0.085	0.039	0.013
Phy (FTU/kg) × Pro (U/kg)					
0 × 0	2.23	4.07	1.85	1.83	0.664
0 × 5000	2.22	4.34	2.12	1.96	0.623
500 × 0	2.17	4.23	2.06	1.95	0.627
500 × 5000	2.16	4.15	1.99	1.93	0.637
*SEM*	0.005	0.119	0.120	0.056	0.019
Phy (FTU/kg) × NMV					
0 × On top base	2.30	4.32	2.01	1.88	0.651
0 × Matrix base	2.14	4.10	1.96	1.91	0.636
500 × On top	2.23	4.24	2.01	1.90	0.644
500 × Matrix base	2.10	4.14	2.05	1.98	0.620
*SEM*	0.005	0.119	0.120	0.056	0.019
Pro (U/kg) × NMV					
0 × On top base	2.31	4.32	2.02	1.88	0.650
0 × Matrix base	2.10	4.99	1.89	1.90	0.640
5000 × On top	2.23	4.24	2.01	1.90	0.644
5000 × Matrix base	2.14	4.25	2.11	1.99	0.615
*SEM*	0.005	0.119	0.120	0.056	0.019
Phy (FTU/kg) × Prot (U/kg) × NMV					
0 × 0 × On top base	2.38^a^	4.29	1.91	1.81	0.675
0 × 0 × Matrix base	2.08^c^	3.86	1.78	1.86	0.652
0 × 5000 × On top base	2.23^b^	4.35	2.12	1.95	0.626
0 × 5000 × Matrix base	2.20^b^	4.33	2.13	1.97	0.620
500 × 0 × On top base	2.23^b^	4.36	2.13	1.95	0.625
500 × 0 × Matrix base	2.11^c^	4.11	2.00	1.95	0.628
500 × 5000 × On top base	2.23^b^	4.13	1.90	1.85	0.662
500 × 5000 × Matrix base	2.08^c^	4.17	2.09	2.00	0.611
*SEM*	0.007	0.168	0.170	0.078	0.026
					*P-value*
Phy	<0.0001	0.906	0.713	0.441	0.539
Pro	0.017	0.435	0.401	0.362	0.415
NMV	<0.0001	0.181	0.925	0.310	0.305
Phy × Pro	0.735	0.150	0.156	0.182	0.183
Phy × NMV	0.013	0.618	0.708	0.715	0.810
Pro × NMV	<0.0001	0.157	0.354	0.587	0.612
Phy × Pro × NMV	<0.0001	0.783	0.728	0.410	0.348

Means in the same column with different superscripts differ significantly (P < 0.05). Each mean represents values from 6 replicates. SEM: standard error of means

*Phy: phytase, &Pro: protease, #NMV: nutritional matrix value. FCE: Feed cost per kg egg mass

## 4 Discussion

In the present study, protease supplementation improved egg production percentage (EP) in laying hens. This improvement may be associated with the enhanced utilization of dietary protein and amino acids resulting from protease activity. Proteases can improve crude protein and amino acid digestibility by degrading anti-nutritional peptides, cleaving peptide bonds and protein complexes, stimulating endogenous peptidase activity, and reducing excessive enzymatic secretion and protein turnover, thereby increasing amino acid availability for protein synthesis and tissue accretion (Freitas et al., 2011; Siegert et al., 2019; Vieira et al., 2013). Among these amino acids, improved ileal digestibility of branched-chain amino acids may be particularly important, as they contribute to the regulation of hepatic fatty acid metabolism and yolk lipoprotein synthesis, which are essential processes for egg formation (Liu et al., 2025; Macelline et al., 2021). In addition, protease supplementation may enhance the digestibility of non-protein nutrients through modifications of the feed nutrient matrix after proteolysis, increased endogenous secretions, and improved intestinal health, as indicated by increased villus height-to-crypt depth ratios in the duodenum and jejunum (Cowieson & Roos, 2016; Ding et al., 2016). These effects may collectively improve nutrient utilization and nutrient deposition into eggs, thereby supporting productive performance in laying hens (Chen et al., 2021; Poudel et al., 2024). Consistent with the present findings, several studies have reported increased EP following dietary protease supplementation in laying hens (Barbosa et al., 2020; Barbosa et al., 2022; Cai et al., 2024; Chen et al., 2021; Liu et al., 2025).

In this study, protease increased EP only in diets without phytase. This suggests that the response to protease may depend on its interaction with other enzymes, such as phytase (Cowieson & Roos, 2013; Vieira et al., 2016). Meta-analytical evidence indicates that the inclusion of phytase or carbohydrases may attenuate the positive effects of protease on amino acid digestibility, suggesting that the efficacy of protease can be diminished when used in multi-enzyme combinations Lee et al. (2018). In line with this, Moss et al. (2017) reported a significant protease × phytase interaction on broiler weight gain, whereby protease supplementation alone enhanced growth performance, whereas its combination with phytase reduced weight gain. Likewise, Walk and Poernama (2019) observed no beneficial effects of protease on broiler performance in diets containing phytase and xylanase. These interactions may arise from overlapping modes of action on protein and amino acid digestion, as both enzymes can reduce endogenous amino acid losses Borda-Molina et al. (2019). A more comprehensive understanding of protease–phytase interactions is critical for precise diet formulation and optimal enzyme efficacy. Therefore, further research in laying hens is warranted.

Protease supplementation caused changes in internal egg quality traits, including HU and YC. The HU is widely recognized as the standard measure of albumen quality and was first developed by Raymond Haugh in 1937 Toomer et al. (2019). This measurement is based on albumen height and thickness, with fresher eggs exhibiting firmer albumen and, consequently, higher HU values Toomer et al. (2019). Several studies have reported that protease supplementation in laying hen diets increases Haugh units (Barbosa et al., 2022; Poudel et al., 2023; Oketch et al., 2025), likely as a result of improved amino acid utilization Oketch et al. (2025). As all basal diets contained comparable levels of corn, the observed improvement in YC is likely attributable to enhanced absorption and deposition of fat-soluble pigments in the yolk, potentially facilitated by protease supplementation (Oketch et al. (2025). This interpretation is supported by the close relationship between lipid digestion and xanthophyll uptake, as improved fat digestibility enhances the intestinal absorption of carotenoid pigments (Freitas et al., 2011). Several studies have reported improvements in fat digestibility following protease supplementation (Fru-Nji et al., 2011; Freitas et al., 2011; Kalmendal & Tauson, 2012). Part of the beneficial effects of protease supplementation on lipid digestibility may be attributed to its ability to degrade anti-nutritional factors in soybean meal, such as lectins, and facilitate the breakdown of complex proteins (da Silva et al., 2026). Moreover, undigested proteins may bind to free fatty acids and interfere with their absorption, whereas enhanced protein hydrolysis may improve lipid utilization (Kalmendal & Tauson, 2012).

Eggshell quality is a critical determinant of commercial egg production efficiency (Carrillo et al., 2020), influenced by both organic matrix components, predominantly proteins, and mineral content (Attia et al., 2020). Adequate dietary Ca is essential for maintaining eggshell integrity and consistency in laying hens (Attia et al., 2020), as calcium deficiency has been shown to adversely affect shell quality parameters (Castillo et al., 2004; Jiang et al., 2013). Phosphorus, another essential and costly nutrient in poultry diets, plays a major role in eggshell formation and mineralization (Pagnussatt et al., 2025). Furthermore, shell formation is also dependent on collagen synthesis, as collagen provides the matrix for calcium deposition and the formation of shell crystals (Barbosa et al., 2022). Maintaining sufficient levels of amino acids, especially sulfur-containing ones, is essential for achieving proper eggshell thickness (Novak et al., 2006). A lack of adequate protein and amino acid supply, however, compromises the integrity of the protein matrix, negatively impacting both the shell’s crystalline structure and its overall thickness (Mazzuco & Bertechini, 2014). Therefore, alterations in nutrient supply can influence EST and shell composition.

In the present study, hens fed diets supplemented with matrix-based phytase exhibited increased EST and improved ESS compared with those receiving on-top phytase supplementation. These improvements are likely associated with higher ash, calcium, and phosphorus deposition in the eggshells of hens fed matrix-based phytase diets compared with those receiving on-top phytase. The enhanced EST observed with phytase supplementation may be attributed to improved access to crucial nutrients, such as minerals and amino acids, within the shell structure (Eltahan et al., 2023; Saleh et al., 2021). On the other hand, adding phytase as an on-top supplement may be associated with changes in the calcium-to-phosphorus balance, which might influence the availability of these minerals for shell development. Calcium and phosphorus absorption are closely linked, so their dietary supply must be balanced and aligned with the bird’s requirements for optimal utilization (David et al., 2023). The ratio of calcium to phosphorus in poultry feed directly affects the digestibility of both minerals (Adedokun & Adeola, 2013). The release of nutrients following phytate breakdown can lead to antagonistic mineral interactions in the gut, hindering absorption (Beeson et al., 2017). An inverse relationship exists between dietary calcium levels and the digestibility of calcium, phosphorus, magnesium, and potassium, along with phytate hydrolysis (David et al., 2023; Paiva et al., 2013; Wilkinson et al., 2011). According to the studies of Mazhar et al. (2025) and Pagnussatt et al. (2025), excess dietary phosphorus in laying hens reduces calcium and phosphorus digestibility and negatively affects eggshell quality. Dijkslag et al. (2021) also reported that higher dietary calcium and phosphorus levels reduced ESS and EST in laying hens at 32 weeks of age. Nutrient oversupply, resulting from high phytase doses, was also shown by Walk et al. (2019) to reduce calcium and phosphorus digestibility in the broiler ileum. Nevertheless, the present findings only demonstrate an association between dietary treatments and eggshell quality traits, rather than establishing a causal mechanism. Further research incorporating direct measurements of nutrient digestibility, calcium and phosphorus retention, and phytate hydrolysis is necessary to elucidate the underlying mechanisms.

The current study also observed that protease supplementation increased eggshell phosphorus content in diets without phytase. The full potential impact of phytase might have been constrained in diets supplemented protease, as the concurrent inclusion of protease could have limited phytase’s effect. Given that most feed ingredients house nutrients within a complex matrix composed of starch, non-starch polysaccharides, proteins, lipids, minerals, and vitamins, feed enzymes are expected to influence nutrients beyond their primary targets (Cowieson & Roos, 2016). Protease supplementation has been shown in various studies to enhance the digestibility of energy (Cowieson & Roos, 2016; Fru-Nji et al., 2011), starch (Kalmendal & Tauson, 2012), fat (Freitas et al., 2011), and minerals like calcium and phosphorus (Walk et al., 2019; Xu et al., 2022), resulting in effects similar to those achieved with phytase. Walk et al. (2019) also confirmed that protease supplementation can improve phosphorus digestibility.

The increase in plasma total protein concentration following protease and phytase supplementation is consistent with the findings of Attia et al. (2025), who reported an increase in plasma total protein concentration with phytase supplementation, and Saleh et al. (2020), who reported an increase in plasma total protein concentration following protease supplementation in broilers. Plasma calcium and phosphorus concentrations were also increased following protease supplementation. Proteases may help mitigate the adverse effects of certain anti-nutritional factors present in soybean meal, such as lectins, which interfere with intestinal epithelial cell proliferation and compromise the integrity of the microvilli brush border membrane, ultimately affecting gut structure, intestinal barrier function, and nutrient absorption (Casaubon-Huguenin et al., 2004; Dosković et al., 2013; Di et al., 2025). Moreover, protease can enhance amino acid availability by improving crude protein digestibility, even in the presence of anti-nutritional factors such as protease inhibitors (Erdaw et al., 2018; Hong et al., 2025; Qiu et al., 2023). In this context, branched-chain amino acids have been shown to promote calcium uptake and reduce its loss via both transcellular and paracellular absorption pathways in the intestines, as well as enhance renal reabsorption (Habibi et al., 2022). Furthermore, non-essential amino acids such as aspartic and glutamic acid — of which soy protein is particularly rich — can form complexes with calcium, thereby improving its absorption efficiency (An et al., 2022). Phosphorus absorption in the jejunum occurs through passive diffusion and active transport via the type IIb sodium–phosphate co-transporter (Saddoris et al., 2010). Inadequate dietary protein or amino acid imbalance reduces the expression of this co-transporter, thereby impairing active phosphorus uptake (Mazhar et al., 2025). Therefore, protease supplementation may contribute to increased phosphorus absorption, possibly through improved amino acid digestibility and a subsequent upregulation of phosphorus transporters (Pagnussatt et al., 2025), although this mechanism remains to be fully established.

The elevated plasma calcium and phosphorus concentrations following phytase supplementation may be attributable to the enzymatic hydrolysis of phytate through the removal of phosphate groups, consequently liberating bound calcium and phosphorus and enhancing their bioavailability for intestinal absorption (Eltahan et al., 2023; Saleh et al., 2021). In line with the findings of the present study, Saleh et al. (2021) found that phytase supplementation at 5000 FTU/kg elevated plasma calcium and phosphorus in laying hens. Mazhar et al. (2025) also observed that phytase at doses of 250 and 500 FYT/kg enhanced plasma calcium and phosphorus concentrations. However, regarding plasma calcium concentration, significant increases were observed in diet with on-top phytase supplementation, whereas elevated plasma phosphorus concentrations were more evident in matrix-based phytase supplementation diet. Under on-top phytase supplementation condition, the enzymatic liberation of phytate-bound phosphorus did not necessarily translate into elevated plasma phosphorus concentrations, which may suggest that birds possess a precise homeostatic regulatory mechanism regulating intestinal phosphorus absorption, with any surplus phosphorus being excreted via feces rather than entering systemic circulation. Moreover, the relatively higher dietary calcium levels associated with on-top phytase supplementation may have compromised phytase efficacy, as elevated calcium concentrations have been demonstrated to inhibit both endogenous and exogenous phytase activity, presumably through the formation of insoluble calcium-phytate complexes (David et al., 2023).

Oxidative stress arises when reactive oxygen species production exceeds cellular antioxidative capacity, leading to lipid peroxidation and impaired antioxidant enzyme activity (Zhang et al., 2021). Overall antioxidant capacity reflects the redox balance between antioxidants and prooxidants, maintained by both enzymatic and non-enzymatic defense systems Surai & Kochish (2019), with MDA serving as a key biomarker of lipid peroxidation and oxidative damage (Zhang et al., 2021). In the present study, matrix-based phytase supplementation increased plasma TAC concentration and resulted in the greatest reduction in plasma MDA concentration. Minerals such as selenium, zinc, and copper, which are often chelated by phytic acid, act as essential cofactors for antioxidant enzymes such as glutathione peroxidase and superoxide dismutase (Zhang et al., 2026). Therefore, phytate hydrolysis enhances their bioavailability and supports antioxidant enzyme activity (Zhang et al., 2026). Mazhar et al. (2025) also observed improved fat digestibility in laying hens receiving 500 FYT/kg of phytase. Efficient fat digestion is essential for the absorption of fat-soluble vitamins, which play a critical role in antioxidant defense (Félix et al., 2020). Furthermore, dietary phosphorus is crucial for mitigating oxidative stress, as its deficiency impairs antioxidant defenses and glutathione synthesis, leading to increased lipid peroxidation (Alagawany et al., 2020; Chen et al., 2017). Nie et al. (2018) reported that increasing dietary non-phytate phosphorus level reduced serum MDA concentrations in laying hens, and a similar effect was observed in geese, where elevating dietary available phosphorus from 0.35% to 0.45% also decreased serum MDA levels, indicating reduced oxidative stress (Alagawany et al., 2020). Although no studies have reported the effects of phytase supplementation on plasma MDA concentrations, Lee et al. (2024) demonstrated reduced MDA levels in the jejunal tissue of broilers, and more recently, Li et al. (2026) reported a reduction in liver MDA concentration in phytase-fed ducks, indicating improved oxidative stability. In contrast, phytase on-top supplementation did not lead to an increase in plasma TAC. This lack of effect may be attributed to higher calcium availability, which can form calcium soaps that impair lipid digestion and bind phytate into insoluble complexes, thereby limiting phytase efficacy and phosphorus absorption. Moreover, higher dietary calcium may increase gut pH, which can negatively affect the absorption of essential trace minerals such as zinc and manganese (David et al., 2023).

The improved redox balance observed with protease addition may be attributed to several potential mechanisms. Protease supplementation may enhance the release of cysteine from dietary proteins, thereby providing a precursor for glutathione synthesis and possibly increasing antioxidant capacity. Improved nutrient digestibility could also enhance the bioavailability of minerals such as selenium, which is important for selenoprotein function. In addition, protease may increase fat digestibility and stimulate endogenous lipase activity, potentially promoting the absorption of fat-soluble vitamins (A and E) that contribute to antioxidant defense (Fru‑Nji et al., 2011; Freitas et al., 2011; Kalmendal & Tauson, 2012; Xu et al., 2022; Yi et al., 2024). Protease supplementation may further influence antioxidant status through modulation of gut microbiota and reduction of intestinal inflammation and oxidative stress (Liu et al., 2024). In addition, it may contribute to the generation of antioxidant bioactive peptides from soybean proteins (Oliveira et al., 2014) and could facilitate the hydrolysis of allergenic proteins such as glycinin and β-conglycinin, which have been suggested to be associated with oxidative stress (Bacou et al., 2021; Park et al., 2020; Yi et al., 2022; Yin et al. 2021; Zhang et al., 2013). Studies by Xu et al. (2022) and Yi et al. (2024) also demonstrated that protease supplementation increases TAC and reduces MDA levels in muscle tissues. Notably, a reduction in plasma MDA concentration was observed in the group fed the protease-on-top supplemented diet, suggesting greater availability of amino acids and a specific effect on lipid peroxidation. Certain amino acids can inhibit lipid peroxidation by scavenging free radicals and chelating metal ions, thereby exhibiting antioxidant activity, with this effect depending on their concentration (Park et al., 2012; Rogulska et al., 2023).

In hens fed diets supplemented with protease, the concentration of LH increased, and the F1 and F2 follicles became larger. The reproductive activity of laying hens is controlled by hormones produced by the hypothalamus–pituitary–gonadal axis Zhao et al. (2023). It is well established that gonadotropins, including FSH and LH, play important roles in follicular growth and maturation and can therefore affect the production performance of laying hens (Zhang et al., 2025). Changes in reproductive hormones can affect the production performance of laying hens (Ding et al., 2024; Niu et al., 2023). Protease enzymes play a crucial role in regulating ovarian functions, including the initiation of follicular development as well as the maturation and ovulation of oocytes (Kushawaha & Pelosi, 2025). In the present study, the increase in plasma LH concentration and the enlargement of F1 and F2 follicles were not accompanied by improvements in all productive performance traits (EW, EM, and FCR). Studies by He et al. (2023), Wu et al. (2024), and Lai et al. (2025) have likewise shown that changes in LH, FSH, and estradiol concentrations are not necessarily associated with improvements in performance traits in laying hens. Eltahan et al. (2023) and Liu et al. (2023) further reported that alterations in reproductive hormones were only associated with an increase in EP, while other performance traits remained unaffected. Collectively, these findings indicate that changes in reproductive hormones (in the present study, LH) do not necessarily translate into consistent improvements across all productive performance parameters. In addition, evidence regarding the effects of phytase and protease supplementation on reproductive hormone profiles in laying hens remains limited. Therefore, the present study may be considered among the early investigations in this area. At this stage, only a tentative association between increased LH concentration and EP following protease supplementation can be proposed, and the underlying mechanisms should be clarified in future research.

The lower feed cost associated with matrix-based phytase supplementation may be related to the adjustment of dietary nutrients according to the phytase matrix values. Although feed cost was affected by dietary treatments, the absence of differences in egg revenue, net profit, and cost–benefit ratio indicates that changes in feed cost alone were insufficient to alter overall economic returns.

## 5 Conclusion

In conclusion, the present study demonstrates that the benefits of phytase and protease supplementation in laying hens depend not only on enzyme inclusion but also on the strategy used for their application. From a practical nutrition perspective, protease supplementation may improve productive performance, particularly in diets without phytase. Furthermore, protease supplementation may positively influence antioxidant status and reproductive-related responses in laying hens. The efficacy of phytase appears to be closely related to the incorporation of its nutrient matrix values into feed formulation. The improvements observed with matrix-based phytase supplementation, particularly in eggshell characteristics and antioxidant status, highlight the importance of accurately applying enzyme matrix values to optimize nutrient utilization rather than simply adding enzymes without dietary nutrient adjustments. Therefore, the application strategy, proper consideration of enzyme matrix values, and potential interactions between phytase and protease should be carefully considered when formulating laying hen diets. Although this study provides new insights into phytase and protease interactions, the underlying mechanisms remain to be fully elucidated. Future studies should investigate nutrient digestibility, mineral retention, phytate degradation, and molecular responses related to nutrient absorption and reproductive regulation. In addition, validation under commercial production conditions is needed to confirm the practical application of enzyme matrix values and develop more effective enzyme supplementation strategies in laying hen nutrition.

## Ethics declaration

This study was conducted in accordance with the ethical standards and animal welfare guidelines of Urmia University and was approved by its Animal Care and Use Committee (Approval No. IR-UU-AEC-3/102).

## CRediT authorship contribution statement

**Elahe Maghami:** Writing – review & editing, Project administration, Methodology, Investigation, Data curation. **Sina Payvastegan:** Writing – review & editing, Writing – original draft, Visualization, Validation, Supervision, Software, Resources, Project administration, Methodology, Investigation, Formal analysis, Data curation, Conceptualization. **Mohsen Daneshyar:** Writing – review & editing, Validation, Methodology, Investigation, Conceptualization. **Touba Nadri:** Writing – review & editing, Methodology, Investigation.

## Declaration of competing interest

The authors confirm that there are no potential conflicts of interest related to this work.
